# High Risk Human Papillomavirus Prevalence in Patients with Hypopharynx Cancer of Northeast India: A Pilot Study

**DOI:** 10.1007/s12070-024-04866-7

**Published:** 2024-07-16

**Authors:** Manigreeva Krishnatreya, Avdhesh Kumar Rai, Mouchumee Bhattacharyya, Tashnin Rahman, Plabita Bhuyan, Anupam Sarma

**Affiliations:** 1 Departments of Cancer Registry and Epidemiology, Bhubaneswar Borooah Cancer Institute, Guwahati, India; 2 DBT Centre for Molecular Biology and Cancer Research, Bhubaneswar Borooah Cancer Institute, Guwahati, India; 3 Departments of Radiation Oncology, Bhubaneswar Borooah Cancer Institute, Guwahati, India; 4 Departments of Head and Neck Surgery, Bhubaneswar Borooah Cancer Institute, Guwahati, India; 5 Departments of Pathology, Bhubaneswar Borooah Cancer Institute, Guwahati, India

**Keywords:** HPV, Hypopharynx cancer, Nested multiplex, PCR

## Abstract

Oncogenic viruses such as, Human papillomavirus (HPV) associated head and neck cancers have been considered to represent different etiological and pathological behaviour. This pilot study was conceived to investigate high risk HPV (hr-HPV) infection and its association with life style habits such as tobacco, alcohol consumption in patients with hypopharynx cancer from North –East India. A total of thirty four primary hypopharynx cancer biopsy specimens were collected. These samples were analysed for hr-HPV DNA using nested multiplex PCR (NMPCR). The lifestyle and dietary associated factors were collected through a self- designed questionnaire. The presence of hr-HPV was confirmed in 50% (*n* = 17) patients with hypopharynx cancer by nested multiplex PCR (NMPCR). Among hr-HPV positive cases, only HPV- 16 genotype was found. Significant association was observed between hr-HPV infections with alcohol consumption (p-0.025), alcohol with tobacco habit (p-0.01). Our study demonstrated that alcohol consumption, tobacco chewing may act as risk factors for hr-HPV infection in a subset of patients with hypopharynx cancer from the North-East region of India.

## Introduction

Cancer is a leading cause of mortality and morbidity worldwide. In developing countries, it is the second leading cause of death and the burden of cancer in these countries is increasing alarmingly. According to the GLOBOCAN 2020 report, there are 19.3 million new cases of cancer and around 10 million deaths from cancer. Because of the large population nearly half of the cancer cases and half of the cancer deaths arise in Asia [1, 2]. According to the reports of the Global Cancer Observatory, there were a total of 1,157,294 new cases of cancer in 2018 and 784,821 deaths in India. The Northeast region of India is known as the cancer hotspot of the country. Population-based cancer registry (PBCR) reports show that the highest cancer incidence is seen in Northeast India where one in every four men and women are likely to develop cancer [3].

The pharynx includes the nasopharynx, oropharynx, and hypopharynx [4]. Head and neck squamous cell carcinoma accounts for approximately 650,555 incident cases and 300,00 deaths annually and is considered the sixth leading cause of cancer mortality [5–7]. Globally, the age-standardized incidence of hypopharynx cancer is highest among both men and women in the region where this study has been undertaken [8].

Cancer of the hypopharynx is characterized by multiple etiological factors like tobacco chewing, cigarette smoking, and alcohol consumption. These 3 factors are synergistic and dose-dependent [9, 10]. However, a subgroup of the population develops oral as well as oropharyngeal cancer without having been exposed to these risk factors, suggesting other etiological factors, such as genetic predisposition, diet, and viral agents — particularly human papillomavirus (HPV) infection [11]. Bosh et al. reported that several human DNA viruses are now accepted as causative factors in approximately 15% of all malignancies worldwide [12].

The correlation of HPV in oral and oropharyngeal carcinogenesis was first proposed by Syrjanen et al. in 1983, and since then many other authors have reported such an association [13–16]. Gillison et al. reported that the high-risk human papillomavirus (hr-HPV) genotypes are involved in the pathogenesis of head and neck squamous carcinoma in the study [17].

Although there is a high incidence of hypopharynx cancer in the region, there is a lack of data on the oncogenic HPV infection status in these cases in the population of NE India. The aim of the present pilot study is to investigate the prevalence of hr-HPV in patients with hypopharynx cancer in NE India and to find its correlation with their dietary and lifestyle-associated factors, if any.

## Materials and Methods

The present study has been approved by the institutional ethics committee of the institute. The study includes patients with hypopharynx cancer diagnosed at a tertiary care cancer center in north east India who were enrolled from November 2021 to March 2022. A total of 34 patients with hypopharynx cancer were enrolled in the study. The tissue samples for identifying hr-HPV were collected at the time of endoscopy and biopsy, which are routine diagnostic procedures in patients with hypopharynx cancer. The tissue samples were collected in 400 µL of 1xPBS (phosphate-buffered saline). Patients were informed about the study and written consent was obtained prior to the collection of the specimens. The lifestyle information was obtained through a self-designed a questionnaire through a personal interview. The information collected includes age, gender, consumption of alcohol, areca nut, tobacco, and other dietary habits (vegetarian and non-vegetarian).

### Genomic DNA Isolation from Tumor Tissue

The genomic DNA was isolated using the phenol-chloroform method. The quantity and quality of the isolated DNA were confirmed by using the Nano-spectrophotometer (Bio-photometer, Eppendorf, Germany) at 260 nm and 0.8% Agarose gel electrophoresis respectively.

### Housekeeping Gene PCR Amplification (β-Actin)

Before HPV-specific amplification, all DNA samples were tested for housekeeping gene amplification (β-actin) to check the presence of amplification inhibitors in the sample. Polymerase chain reaction (PCR) was done in 10 µL volume using 50-100ng of genomic DNA, 2x EmeraldAmp MAX PCR Master Mix, 10 µM primers, and 5%DMSO. The thermal cycling condition was as: Denaturation- 95 °C for 5 min, 40 amplification cycles with Denaturation-94 °C for 30 s, Annealing- 52 °C for 60 s and Extension- 72 °C for 90 s, and final extension 72 °C for 10 min. All PCR products were visualized in 2% agarose gel (Amresco, USA) stained with ethidium bromide (Amresco, USA) in the Gel Doc XR™ system (Bio-Rad, USA).

### HPV Detection by E6 Nested Multiplex PCR (NMPCR)

The isolated DNA was then further analyzed for the presence of HPV. The nested PCR reaction 1st round was done in 20 µL volume using 100ng genomic DNA, 2x EmeraldAmp MAX PCR Master Mix, 10 µM primers, and 6.5%DMSO. The thermal cycling condition for the 1st round of PCR was as: Denaturation- 95 °C for 5 min, 38 amplification cycles with Denaturation-95 °C for 30 s, Annealing- 52.5 °C for 60 s and Extension- 72 °C for 90 s and final extension 72 °C for 10 min. The 1st round PCR product (630 bp) was now used as a template for the 2nd round of PCR reaction using type-specific HPV primers. The thermal conditions for 2nd round nested PCR conditions for HPV genotyping were similar to the 1st round with changes only in Annealing- 56.5 °C–40 s and Extension-72 °C–45 s. The hr-HPV positive cervical squamous cell carcinoma samples DNA was used as positive control and a negative control containing sterile water was used in each PCR run. All PCR products were then visualized in 2% agarose gel (Amresco, USA) which is stained with ethidium bromide (Amresco, USA) in the Gel Doc XR™ system (Bio-Rad, USA). The primer sequences are described in Table 1.

**Table 1:** Primer sequence for HPV type specific nested multiplex PCR

Primers		Primer Sequence (5’ to 3’)	Amplicon size (bp)	
GP-E6-3 F		Sense: GGG AGG TAC TGA AAT CGG T	630	
GP-E6-6B		Antisense: TCC TCT GAG TCG CCT AAT TGC TC		
**HPV type-specific Nested PCR Primer Sequences**				
High risk- HPV genotype	**Primer Sequence (5’ to 3’)**			**Amplicon size (bp)**
HPV-16	Sense: CAC AGT TAT GCA CAG AGC TGC			457
	Antisense: CAT ATA TTC ATG CAA TGT AGG TGT A			
HPV-18	Sense: CAC TTC ACT GCA AGA CAT AGA			322
	Antisense: GTT GTG AAA TCG TCG TTT TTC A			
HPV-31	Sense: GAA ATT GCA TGA ACT AAG CTC G			263
	Antisense: CAC ATA TAC CTT TGT TTG TCA A			
HPV-59	Sense: CAA AGG GGA ACT GCA AGA AAG			215
	Antisense: TAT AAC AGC GTA TCA GCA GC			
HPV-45	Sense: GTG GAA AAG TGC ATT ACA GG			151
	Antisense: ACC TCT GTG CGT TCC AAT GT			

### Statistical Analysis

The hr-HPV prevalence was shown according to different demographic characteristics and Chi-square tests were used to compare the differences of hr-HPV prevalence between different groups. *P* < 0.05 was considered as statistically significant. SPSS ver29 was used for all the statistical analyses.

## Results

Out of the total (*n* = 34) patients, 88% (*n* = 30) of patients with hypopharynx cancer were men and the rest 12% (*n* = 4) were women. The mean age of the sample group was 56 (range: 27–85).

Non-vegetarian diet was reported in 70.5% (*n* = 24) of the patient population and the rest 29.5% (*n* = 10) followed a vegetarian diet. Alcohol consumption was found in 67.6% (*n* = 23), tobacco consumption in 70.5% (*n* = 24), betel nut chewers was found in 70.5% (*n* = 24), and smoking habit in 58.8% (*n* = 20) patients with hypopharynx cancer. Alcohol and chewing tobacco habit was found in 64.7% (*n* = 22) patients with hypopharynx cancer and both alcohol and smoking habit in 70.5% (*n* = 24) of the total study cohort. The details of the dietary and alcohol/tobacco/areca nut consumption are given in Table 2 for both hr-HPV.

**Table 2:** Demographic profile in association with hr-HPV status in hypopharyngeal cancer patients

Characteristic	Total Cases	hr-HPV positive	hr-HPV negative	*P*-value
**Gender**				
Male Female	30 4	15 2	15 2	1.000
***Age***				
< 40 > 40	5 29	1 16	4 13	0.335
***Dietary habits***				
Vegetarian Non-vegetarian	10 24	2 11	8 13	0.258
***Alcohol intake***				
No-intake Intake	11 23	4 15	7 8	0.0255
***Tobacco-chewers***				
Non-chewers Chewers	10 24	8 11	2 13	0.128
***Areca nut***				
Non-chewers Chewers	10 24	8 11	2 13	0.128
***Smoking habits***				
Non-smokers Smokers	14 20	6 13	8 7	0.296
***Both alcohol-tobacco*** ***Habits***				
Non-intake Intake	12 22	5 12	7 10	0.720
***Both alcohol-smoking*** ***habits***				
Non-intake Intake	10 24	3 14	7 10	0.258

Squamous cell carcinoma was the only histological type found in the present study population. Moderately differentiated squamous cell carcinoma was the predominantly found morphological feature (66%), followed by well-differentiated squamous cell carcinoma (22%) and poorly differentiated squamous cell carcinoma (12%).

Housekeeping gene amplification (256 bp) bands were observed in all the samples (*n* = 34). High-risk- HPV was detected by the Nested Multiplex PCR in 50% (*n* = 17) of the total study sample. HPV16 was the only high-risk HPV genotype that was detected in the sample population.

The study population was grouped into the ‘HPV positive’ and ‘HPV negative’ based on the presence of hr-HPV DNA and their association with various factors such as age, gender, dietary, alcohol, and tobacco habits are analyzed (Table 2). A significant association was found between hr-HPV infection and alcohol intake (*p* = 0.0255) and no such association was found between other factors such as age, gender, tobacco, areca nut, etc.

## Discussion

HPV is now a well-established etiological factor for the development of HNCs particularly oropharynx squamous carcinoma [18]. In our study, hr-HPV was detected in 50% (*n* = 17) of the patients with hypopharynx cancer. And HPV 16 is the only high-risk HPV genotype type that was found in the studied patient population. Our result is supported by a previous study done in India, in which hr-HPV DNA was found in 23–73% of oral cancer cases [19–21]. As per a recent study on the Northeast Indian population, hr-HPV is significantly associated with oropharynx cancer [22], which is an adjacent anatomical site of the hypopharynx. The difference may be attributed to primarily the tumor site which is the hypopharynx in the present study and other variety of factors such as different methodologies, PCR sensitivities, low sample size, etc.

Our study demonstrates that alcohol consumption is significantly associated with hr-HPV positivity in hypopharynx cancer. This observation is in concordance to previous studies, which showed that alcohol intake is associated with a significantly higher risk of HPV infection [23]. Other etiological factors such as tobacco, areca nut, etc. were not found to be significantly associated with hr-HPV positivity in patients with hypopharynx cancer.

Our preliminary finding needs confirmatory analysis by a case-control study design. Furthermore, there is relatively poor 5-year overall survival (36.9%) among patients with hypopharynx cancer in the region along with the highest age-standardized incidence [8, 24], which warrants risk prognostication with hr-HPV in this group of patients. A recent study from Japan showed hr-HPV positivity in hypopharyngeal cancer at 0.4% or extremely rare [25]. However, our study showed a very high prevalence (50%) of hr-HPV in patients with squamous carcinoma of the hypopharynx in our population. This is a significant finding.

### Limitations of the Study

This is a pilot study with a very small sample size and with its inherent limitations. This type of study can show the association of hr-HPV infection in a subset of patients with hypopharynx cancer and not its causation.

### Strength of the Study

This is the first-of-its-kind pilot study from the highest-risk region of the world for patients with hypopharynx cancer. This study can potentially open a new vista for prevention and risk prognostication for patients with hypopharynx cancer.

## Conclusion

The present pilot study showed that hr-HPV infection is prevalent in alcohol-consuming and tobacco-chewing patients with hypopharynx cancer. Additionally, hr-HPV infection may be a risk factor for the development of hypopharynx cancer in the population of North-East India.
